# Biofortification of Six Varieties of Lettuce (*Lactuca sativa* L.) With Iodine and Selenium in Combination With the Application of Salicylic Acid

**DOI:** 10.3389/fpls.2019.00143

**Published:** 2019-02-19

**Authors:** Sylwester Smoleń, Iwona Kowalska, Peter Kováčik, Mariya Halka, Włodzimierz Sady

**Affiliations:** ^1^Unit of Plant Nutrition, Institute of Plant Biology and Biotechnology, Faculty of Biotechnology and Horticulture, University of Agriculture in Krakow, Kraków, Poland; ^2^Department of Agrochemistry and Plant Nutrition, Slovak University of Agriculture in Nitra, Nitra, Slovakia

**Keywords:** iodine, selenium, salicylic acid, biofortification, proline, Se-amino acid, varieties of lettuce

## Abstract

The agrotechnical methods of biofortification of plants, i.e., enriching them in iodine (I) and selenium (Se) could be effective methods to enrich food products in these elements. The advantage of agrotechnical methods of biofortification is the incorporation of elements in organic compounds in plants; therefore, they have better health-promoting properties than pure technical salts. Two-year studies were conducted in a greenhouse with hydroponic cultivation of three botanical varieties of lettuce in an NFT (nutrient film technique) system: two cultivars butterhead lettuces (abb. BUTL) ‘Cud Voorburgu’ and ‘Zimująca,’ two cultivars iceberg lettuces (abb. ICEL) ‘Maugli’ and ‘Królowa lata’ (all this four cultivars are classified as *Lactuca sativa* L. var. *capitata*) as well two cultivars *Lactuca sativa* L. var. *crispa* L. cultivars (abb. REDL) ‘Lollo rossa’ and ‘Redin’ having little red leaves. The study included the application of I (as KIO_3_), Se (as Na_2_SeO_3_), and SA into the nutrient solution. The tested treatments were as follows: (1) control, (2) I+Se, (3) I+Se+0.1 mg SA dm^−3^, (4) I+Se+1.0 mg SA dm^−3^, and (5) I+Se+10.0 mg SA dm^−3^. KIO_3_ was used at a dose of 5 mg I dm^−3^, while Na_2_SeO_3_ was 0.5 mg Se dm^−3^. Regardless of the kind of the applied compound, the highest biomass of heads was produced by the REDL ‘Redin’ variety. Furthermore, this variety, as the only one in six varieties tested, reacted with the decrease in yield to the application of I+Se and I+Se+three concentrations of SA. In the heads of all cultivars, the level of I accumulation was 10–30 times higher than of Se. The level of I accumulation formed the following order: REDL ‘Lollo rossa’ > REDL ‘Redin’ = BUTL ‘Cud Voorburgu’ > BUTL ‘Zimująca’ > ICEL ‘Maugli’ > ICEL ‘Królowa lata’. The order of Se content in leaves was as follows: REDL ‘Redin’ = BUTL ‘Cud Voorburgu’ > REDL ‘Lollo rossa’ > ICEL ‘Maugli’ > BUTL ‘Zimująca’ > ICEL ‘Królowa lata’. The obtained results indicate that the introduction of SA to the nutrient solutions in hydroponic systems may allow an improve the effectiveness of – biofortification.

## Introduction

Because of a widespread deficit of various mineral components in the human diet and in animal fodders, the studies on biofortification (enriching) cultivated plants in various elements are undertaken everywhere (García-Bañuelos et al., 2014; Krzepiłko et al., 2016; Przybysz et al., 2016a,b; Żuk-Gołaszewska et al., 2016; Hegedüsová et al., 2017). The effect of iodine iodides and iodates on basic physiological and biochemical processes in lettuce grown in hydroponics and soilless culture was tested (Blasco et al., 2010b; Voogt and Jackson, 2010; Voogt et al., 2010). Similar effect of selenates and selenites on, e.g., biomass, antioxidant activity, the content of secondary metabolites in lettuce plants was also tested (Ríos et al., 2008, 2010).

The agrotechnical method of biofortifying plants with mineral components are regarded as one of the cheapest ways leading to the reduction of their deficits in the human diet and in animal fodders (Zhao and McGrath, 2009; Dayod et al., 2010; Korobova, 2010). In the cases of iodine (I) and selenium (Se), the deficits of these elements pertain to approximately 30 and 15% of the world human population, respectively (White and Broadley, 2009).

In the last few years, a number of research projects have been carried out, aiming at working out the agrotechnical methods of biofortification (fertilizing) of plants, separately with I (Cakmak, 2008; Lawson et al., 2015; Li et al., 2017) or Se (Ríos et al., 2008, 2010; Hawrylak-Nowak et al., 2015; Golubkina et al., 2017). The issues pertaining to the interaction between I and Se during simultaneous fertilizing with these elements are not sufficiently known. Both these elements are not essential for the proper functions of plant bodies. Se and I are, nevertheless, classified in the group of “beneficial elements” for plants (Kopsell and Kopsell, 2007; Medrano-Macías et al., 2016; Gonzali et al., 2017).

For many years, the hydroponic system NFT (nutrient film technique) has been used in the studies on the mineral nutrition of plants. The NFT proves perfectly correct in the studies on lettuce (Rożek et al., 1989; Sady et al., 1990). One of the advantages of the system is the easiness in collecting samples of plant leaves and roots for research, without fear of their contamination with soil, as is common in cultivars growing in the soil, peat or in other substrates. Furthermore, the effectiveness of enriching the plants in mineral components (including trace elements, e.g., I and Se) in hydroponic and/or soilless cultures is higher than that in soil cultures (Dai et al., 2006; Ríos et al., 2010; Voogt et al., 2010). It results from the fact that in the hydroponics the processes of sorption or the processes of the changes in the speciation forms of elements do not occur. These processes are characteristic to the soil environment. They can limit the availability of mineral components to plants and, as a consequence, their uptake by roots.

In hydroponic cultures, there is a problem of the easy spread of fungal and bacterial diseases with the nutrient solution, particularly when the cultures are not equipped with solution disinfection systems. In the hydroponic system with the recirculation of nutrient solution, there is often an increasing level of EC (Electrical Conductivity) – increased degree of nutrient solution salinity. Introducing salicylic acid (SA) to the media in hydroponic and soilless cultures can perform manifold functions. The acid can cause the increase in resistance to abiotic and biotic stress factors (including pathogens) (Hayat et al., 2010). The results of numerous studies demonstrated that SA increases the resistance of various plant species to fungal diseases (Spletzer and Enyedi, 1999; Tari et al., 2002; Mandal et al., 2009). The SA added to media can also increase the uptake of mineral components by plants, including iodine (Smoleń et al., 2015, 2016).

Until now, no studies have been conducted aiming at the determination of the effectiveness of combined biofortification in I and Se in various varieties of lettuce. Earlier such studies were done among others, on pea (Jerše et al., 2018) or kohlrabi sprouts Osmić et al. (2017). Moreover, no studies have been done to date on the determination of various doses of exogenous SA upon the intake and distribution of I and Se (with their simultaneous application) by various varieties of lettuce. The presented study fills in the information gap in this area of knowledge.

The goal of this study was to determine the influence of exogenous SA on the process of three botanical varieties of lettuce (belonging to three types of botanical lettuce) plant biofortification simultaneously with I and Se in a hydroponic NFT system.

## Materials and Methods

### Plant Material and Treatments

Two-year studies were conducted in a greenhouse with hydroponic cultivation of three botanical varieties of lettuce cultivars in an NFT (nutrient film technique) system: two cultivars butterhead lettuces (abb. BUTL) ‘Cud Voorburgu’ and ‘Zimująca,’ two cultivars iceberg lettuces (abb. ICEL) ‘Maugli’ and ‘Królowa lata’ (all this four cultivars are classified as *Lactuca sativa* L. var. *capitata*), as well as two cultivars *Lactuca sativa* L. var. *crispa* L. cultivars (abb. REDL) ‘Lollo rossa’ and ‘Redin’ having little red leaves.

The experiment was conducted in a greenhouse of the Faculty of Biotechnology and Horticulture, University of Agriculture in Kraków, Poland. Each year, seeds were sown into mineral wool plugs (Grodan, Rockwool B.V., Roermond, Netherlands) at the end of August and the beginning of September. Seedlings at the 2-leaf stage were placed into holes (spaced 25 cm apart) of Styrofoam slabs filling NFT beds (‘dry hydroponic’ method). No additional substrate was used. The greenhouse was equipped with five individual NFT sets with 1,300-dm^3^ nutrient solution containers, facilitating lettuce cultivation in recirculating hydroponics.

After plant seedlings were planted in the hydroponic systems, day and night temperatures were set to 15 and 10°C, respectively. From the beginning of October to the end of the experiment, natural light was supplemented between 5.00 and 10.00 a.m. with the use of 600-W high-pressure sodium lamps.

The study included the application of I (as KIO_3_, p.a., Avantor Performance Materials, Gliwice, Poland), Se (as Na_2_SeO_3_, puriss. p.a., Sigma-Aldrich, St. Louis, MO, United States) and SA (puriss. p.a., Avantor Performance Materials) into the nutrient solution to cultivation all types of cultivars. The tested treatments were as follows: (1) control (trace I and Se levels in the nutrient solution from applied fertilizers; 30 μg ⋅ dm^−3^ I and 8.5 μg ⋅ dm^−3^ Se, respectively), (2) I + Se, (3) I + Se + 0.1 SA (0.1 mg SA ⋅ dm^−3^ nutrient solution; i.e., 0.724 μM SA), (4) I + Se + 1.0 SA (1.0 mg SA ⋅ dm^−3^ nutrient solution; i.e., 7.24 μM SA), and (5) I + Se + 10.0 SA (10.0 mg SA ⋅ dm^−3^ nutrient solution; i.e., 72.4 μM SA). KIO_3_ was used at a dose of 5 mg I ⋅ dm^−3^ (i.e., 39.4 μM I), while Na_2_SeO_3_ was at 0.5 mg Se ⋅ dm^−3^ (i.e., 6.3 μM Se). I, Se, and SA were instantly introduced into the nutrient solution beginning at the 3-to-4-leaf stage (formation of the rosette). The experiment was conducted according to a randomized block design with four replications–three plants per one replicate in each treatment. Plants were grown in a nutrient solution with pH 5.50, EC 1.8 mS ⋅ cm^−1^ and the following contents of macro- and micronutrients (mg ⋅ dm^−3^): 120 N, 40 P, 170 K, 35 Mg, 150 Ca, 1.5 Fe, 0.55 Mn, 0.25 Zn, 0.2 B, 0.09 Cu, and 0.04 Mo, which is equivalent to 8.57 mM N, 1.29 mM P, 4.35 mM K, 1.44 mM Mg, 3.74 mM Ca, 26.9 μM Fe, 10.0 μM Mn, 3.8 μM Zn, 18.5 μM B, 1.4 μM Cu, and 0.4 μM Mo.

For each treatment, 1,300 dm^3^ of nutrient solution were stored in separate containers and periodically administered to the cultivation slabs. The frequency of watering was adjusted for the growth stage of lettuce and weather conditions. Plants were cultivated in the recirculating system of nutrient solution without a disinfection system. Plants used the same nutrient solutions throughout the entire period.

Lettuce harvest, followed by the assessment of head and root weight and collection of leaf and root samples, was conducted at the beginning of December in each year of the study.

### Plant Analysis

In this chapter, only a general outline of the analytical methods is presented: selenomethionine, selenocysteine, proline, and SA with the application of PA 800 Plus capillary electrophoresis system Beckman Coulter. The methods and conditions in which the analyses were made are identical to those applied in our earlier publication (Smoleń et al., 2016), where more details are provided.

For the analyses described in this subchapter, lettuce heads were cut in half and mixed in order to obtain a representative sample of all leaves (old and young) from all heads in each treatment.

Samples of fresh lettuce roots and leaves (after being washed in distilled water) were dried at 70°C in a laboratory dryer with forced air circulation and ground in a FRITSCH Pulverisette 14 variable speed rotor mill (Idar-Oberstein, Germany) using a 0.5-mm sieve. Samples were subsequently analyzed with respect to the contents of the following elements: I and Se in leaves and roots by the ICP-OES technique.

In root and leaf samples, the following analysis was also performed: selenomethionine (SeMet; Acros Organics, Geel, Belgium), selenocysteine (SeCys; Sigma-Aldrich), proline (Sigma-Aldrich), and SA (puriss. p.a., Avantor Performance Materials) using capillary electrophoresis via a PA 800 Plus system (Beckman Coulter).

The determination of the I and Se content in root and leaf samples after tetramethylammonium hydroxide (TMAH) extraction was as follows: amounts of 0.5 g air-dried leaf or root samples, 10 cm^3^ double-distilled water and 1 cm^3^ of 25% TMAH (Sigma-Aldrich) were added to 30-cm^3^ Falcon tubes. After mixing, samples were incubated for 3 h at 90°C. After incubation, samples were cooled to a temperature of approximately 20°C and filled to 30 cm^3^ with double-distilled water. After mixing, samples were centrifuged for 15 min at 4,500 rpm. The measurements of I and Se content using an ICP-OES spectrometer (Leeman Labs) were conducted in the supernatant without decanting (PN-EN 15111:2008, 2008).

### Data Analysis

The data were subjected to variance analysis using the of two way analysis of variance (treatments/this is a type of nutrient solutions/ × variety of lettuce) (ANOVA) module of Statistica 10.0 PL software. It was decided to verify whether the tested factors had a significant influence on lettuce biomass and the analyzed parameters of the chemical composition of the plants. The Duncan test was used to determine the significance between the means at a level of *P* < 0.05.

#### Biofortification Target

The percentage of recommended daily allowance for I (RDA-I) and Se (RDA-Se) supplied from one serving of 50 g fresh lettuce leaves was calculated using the results of I and Se content in fresh lettuce leaves as well as the recommended daily intake of these two elements for adults: 150 μg I and 55 μg Se daily (Institute of Medicine, 2000; Andersson et al., 2007).

## Results

**FIGURE 1:**
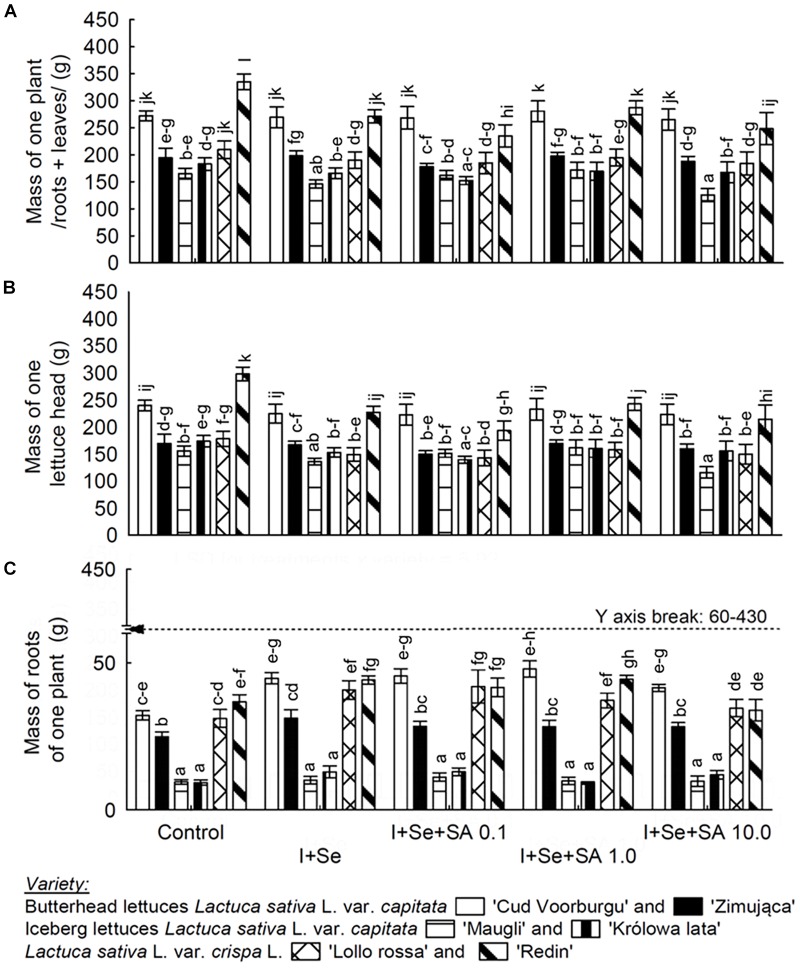
Effects of I, Se, and salicylic acid (SA) application on the whole biomass/roots + leaves per plant **(A)**, mass of a single lettuce head **(B)** and roots per plant **(C)**. Means followed by different letters for “Treatments × Variety” differ significantly at *P* < 0.05. Bars indicate standard error (*n* = 8).

**FIGURE 2:**
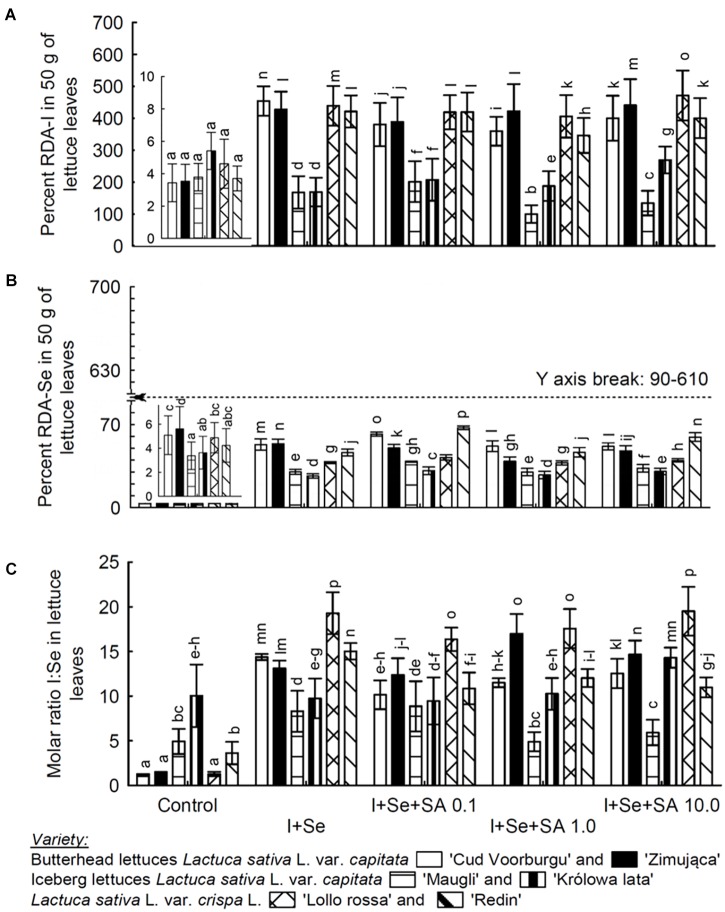
Value percentage of recommended daily allowance (RDA) for I **(A)** and Se **(B)** in a 50-g portion of fresh lettuce and the I:Se molar mass ratio in lettuce leaves **(C)**. **(A,B)** Also shows the enlargement of the bars in the graph for control plants. Means followed by different letters for “Treatments × Variety” differ significantly at *P* < 0.05. Bars indicate standard error (*n* = 8).

The varieties of lettuce showed statistically significant and different responses toward the application of I+Se, and SA, in terms of the quantity of biomass (Figure 1A–C), and in terms of the content of I, Se, SeMet, SeCys, SA, and proline in leaves and roots (Tables 1–3), and the percentage RDA-I and RDA-Se as well as the molar ratio I:Se in lettuce leaves (Figure 2A–C).

**Table 1:** Concentrations of I and Se in leaves and roots of lettuce.

Treatments	Variety	mg I ⋅ kg^−1^ d.w.		mg Se ⋅ kg^−1^ d.w.	
		Leaves	Roots	Leaves	Roots
Control	‘Cud Voorburgu’	2.2 ± 0.74a	9.4 ± 2.16a	1.2 ± 0.37a	2.2 ± 0.51a
	‘Zimująca’	2.0 ± 0.61a	12.7 ± 2.93a	1.2 ± 0.39a	3.4 ± 0.46a
	‘Maugli’	3.2 ± 0.71a	30.1 ± 6.87ab	1.0 ± 0.35a	12.8 ± 4.53ab
	‘Królowa lata’	4.1 ± 0.93a	53.7 ± 13.58b	1.0 ± 0.39a	17.5 ± 4.60b
	‘Lollo rossa’	3.2 ± 1.03a	11.7 ± 3.52a	1.3 ± 0.31a	3.1 ± 0.72a
	‘Redin’	2.5 ± 0.55a	11.8 ± 3.31a	1.1 ± 0.36a	2.9 ± 0.21a
I+Se	‘Cud Voorburgu’	252.4 ± 26.17o	212.7 ± 27.40c	10.8 ± 0.96jk	134.7 ± 1.21c–f
	‘Zimująca’	206.2 ± 41.75i	234.5 ± 24.57c–e	9.7 ± 0.89hi	124.4 ± 3.08c
	‘Maugli’	114.8 ± 32.81d	238.8 ± 45.69c–e	8.2 ± 0.21d	130.8 ± 6.81cd
	‘Królowa lata’	123.1 ± 34.03de	271.4 ± 31.46e–g	7.8 ± 0.41bc	155.3 ± 1.86i–l
	‘Lollo rossa’	292.3 ± 37.97r	314.2 ± 78.16gh	9.4 ± 0.12fg	138.8 ± 17.06d–g
	‘Redin’	245.0 ± 31.65n	245.2 ± 37.20c–g	9.3 ± 0.71fg	138.4 ± 4.98d–g
I+Se+SA 0.1	‘Cud Voorburgu’	218.2 ± 38.45jk	240.8 ± 22.77c–f	13.1 ± 0.30m	163.4 ± 6.81L–m
	‘Zimująca’	218.1 ± 31.76j	259.7 ± 19.49d–g	10.4 ± 0.65jk	161.9 ± 6.57k–m
	‘Maugli’	135.9 ± 39.86e	237.4 ± 31.00c–e	11.1 ± 0.72k	168.5 ± 1.09m
	‘Królowa lata’	153.9 ± 46.53f	279.4 ± 29.46g	9.1 ± 0.53ef	185.0 ± 6.34n
	‘Lollo rossa’	288.8 ± 34.68r	258.3 ± 25.69d–g	10.7 ± 0.47j	141.3 ± 5.37d–h
	‘Redin’	236.3 ± 36.97m	227.8 ± 28.72cd	13.7 ± 0.15n	149.4 ± 2.80g–j
I+Se+SA 1.0	‘Cud Voorburgu’	201.6 ± 25.87hi	226.1 ± 26.03cd	10.6 ± 0.97j	145.6 ± 4.81f–j
	‘Zimująca’	224.7 ± 46.91kl	233.7 ± 33.65c–e	7.6 ± 0.74bc	136.2 ± 4.31c–f
	‘Maugli’	75.1 ± 18.60b	222.5 ± 44.99cd	8.9 ± 0.42de	156.3 ± 15.04i–l
	‘Królowa lata’	132.1 ± 28.93e	242.9 ± 32.55c–g	7.5 ± 0.52b	187.0 ± 7.27n
	‘Lollo rossa’	264.2 ± 39.47pr	245.5 ± 44.02c–g	9.1 ± 0.28ef	132.7 ± 8.71c–e
	‘Redin’	195.6 ± 31.57h	216.3 ± 34.41c	9.9 ± 0.85g–i	151.1 ± 10.86h–k
I+Se+SA 10.0	‘Cud Voorburgu’	223.8 ± 36.73kl	216.5 ± 25.45c	10.7 ± 0.45j	141.1 ± 1.43d–h
	‘Zimująca’	226.5 ± 37.34l	235.3 ± 32.40c–e	9.2 ± 0.62ef	133.0 ± 4.02c–e
	‘Maugli’	94.2 ± 25.09c	247.1 ± 40.57c–g	9.2 ± 0.42ef	145.1 ± 3.90e–i
	‘Królowa lata’	182.8 ± 26.37g	269.9 ± 35.16e–g	7.7 ± 0.57bc	157.5 ± 1.60j–m
	‘Lollo rossa’	304.7 ± 42.87s	277.7 ± 54.57f–g	9.7 ± 0.11g–i	142.0 ± 13.85d–h
	‘Redin’	215.2 ± 30.72j	247.3 ± 45.72c–g	11.8 ± 0.57l	154.1 ± 11.32i–l

*Means in the column followed by different letters for “Treatments × Variety” differ significantly at P < 0.05 (n = 8). d.w., dry weight.*

### Lettuce Biomass

Among the six lettuce varieties, only REDL ‘Redin’ was characterized by a negative response expressed in the amount of biomass toward adding I+Se and I+Se to the nutrient solution in combination with SA (in three different dosages) (Figure 1A–C). In comparison with the control, applying I+Se alone to the nutrient solution and the additional application of SA resulted in significantly decreasing the amount of head and root mass, as well as the mass of whole plants of the REDL ‘Redin’ variety. In the ICEL ‘Maugli’ variety, only combined application of I+Se+SA in the highest dose of 10 mg SA ⋅ dm^−3^ (compared with the remaining types of media under study) caused a significant decrease in masses of heads and whole plants (roots + leaves/heads/).

Concerning the remaining varieties (BUTL ‘Cud Voorburgu,’ ‘Zimująca,’ ICEL ‘Królowa lata,’ and REDL ‘Lollo rossa’), the applied combinations of media with I, Se, and SA, compared with the control, had no effect on the amount of biomass of roots, heads, and whole plants (Figure 1A–C).

### Content of I, Se, and Se-Amino Acid in Lettuce Plants

Adding I and Se to the nutrient solution resulted in a significant increase in the content of these elements in leaves and roots of all varieties of lettuce (Table 1). The I contents in leaves and roots were at a similar order of magnitude, while the Se concentration in roots was approx. ten times higher than that in leaves. Furthermore, the I content in leaves was from 10 to 30 times higher than the Se content.

Comparing the varieties of lettuce in terms of their capacities to accumulate I and Se in leaves indicates that the levels of accumulation of iodine formed the following order: REDL ‘Lollo rossa’ > REDL ‘Redin’ = BUTL ‘Cud Voorburgu’ > BUTL ‘Zimująca’ > ICEL ‘Maugli’ > ICEL ‘Królowa lata’ (Table 1). Whereas the Se content in leaves formed the following order: REDL ‘Redin’ = BUTL ‘Cud Voorburgu’ > REDL ‘Lollo rossa’ > ICEL ‘Maugli’ > BUTL ‘Zimująca’ > ICEL ‘Królowa lata’. Therefore, both varieties of REDL demonstrated the capacities to accumulate I and Se in the greatest amounts, while ICEL ‘Królowa lata’ was characterized by the lowest level of the accumulation of these elements in leaves. It is important that both varieties of REDL showed the lowest content of SeMet and SeCys in leaves simultaneously (Table 2). Next, the highest content of both of these Se-amino acids were noted in the ICEL ‘Królowa lata’ and BUTL ‘Zimująca’ varieties.

**Table 2:** Concentrations of selenomethionine (SeMet) and selenocysteine (SeCys) in leaves and roots of lettuce.

Treatments	Variety	mg SeMet ⋅ kg^−1^ d.w.		mg SeCys ⋅ kg^−1^ d.w.	
		Leaves	Roots	Leaves	Roots
Control	‘Cud Voorburgu’	1.47 ± 0.15a–c	1.24 ± 0.33a	0.54 ± 0.07a–c	0.64 ± 0.10a–c
	‘Zimująca’	1.93 ± 0.62a–d	0.80 ± 0.30a	0.79 ± 0.15b–f	0.36 ± 0.14a
	‘Maugli’	1.52 ± 0.44a–c	0.90 ± 0.12a	0.42 ± 0.05a	0.46 ± 0.06ab
	‘Królowa lata’	3.73 ± 1.24f–j	1.69 ± 0.12a	0.72 ± 0.19a–e	0.63 ± 0.07a–c
	‘Lollo rossa’	1.38 ± 0.54ab	1.18 ± 0.45a	0.46 ± 0.19ab	0.38 ± 0.15a
	‘Redin’	0.83 ± 0.32a	1.26 ± 0.49a	0.35 ± 0.14a	0.38 ± 0.15a
I+Se	‘Cud Voorburgu’	4.28 ± 0.53g–n	3.19 ± 0.18ef	2.07 ± 0.23l	1.70 ± 0.15i–k
	‘Zimująca’	3.91 ± 0.55f–k	4.05 ± 0.25h–k	1.66 ± 0.13g–k	2.18 ± 0.09m
	‘Maugli’	4.46 ± 0.67h–m	5.56 ± 0.93l	1.71 ± 0.17g–l	1.86 ± 0.18kl
	‘Królowa lata’	5.36 ± 1.22mn	2.95 ± 0.34de	1.59 ± 0.20g–i	1.55 ± 0.09ij
	‘Lollo rossa’	2.65 ± 1.03b–f	2.81 ± 1.07c–e	0.88 ± 0.34c–e	1.03 ± 0.39d–g
	‘Redin’	3.35 ± 1.28e–h	2.70 ± 1.03cd	0.60 ± 0.24a–c	0.81 ± 0.31c–e
I+Se+SA 0.1	‘Cud Voorburgu’	4.17 ± 0.31g–m	3.25 ± 0.22ef	2.04 ± 0.25kl	2.49 ± 0.14n
	‘Zimująca’	5.06 ± 0.53k–m	3.21 ± 0.10ef	1.95 ± 0.12i–l	2.03 ± 0.24lm
	‘Maugli’	3.71 ± 0.47e–j	4.31 ± 0.64jk	1.75 ± 0.16g–l	1.91 ± 0.16k–m
	‘Królowa lata’	5.27 ± 0.73mn	3.66 ± 0.60f–h	1.87 ± 0.11h–l	1.22 ± 0.12gh
	‘Lollo rossa’	2.44 ± 0.94b–e	2.88 ± 1.10de	0.69 ± 0.27a–d	1.14 ± 0.43fg
	‘Redin’	3.10 ± 1.17d–g	3.90 ± 1.47g–j	0.88 ± 0.35c–e	1.09 ± 0.43e–g
I+Se+SA 1.0	‘Cud Voorburgu’	5.22 ± 0.56mn	3.67 ± 0.24f–h	1.49 ± 0.22gh	2.04 ± 0.18lm
	‘Zimująca’	4.88 ± 0.41j–m	4.22 ± 0.29i–k	1.98 ± 0.06L	2.61 ± 0.21n
	‘Maugli’	5.08 ± 0.87k–n	3.66 ± 0.76f–h	1.76 ± 0.22g–l	1.63 ± 0.08i–k
	‘Królowa lata’	4.43 ± 0.84h–n	3.82 ± 0.63g–i	1.89 ± 0.14j–l	1.49 ± 0.08hi
	‘Lollo rossa’	3.38 ± 1.28e–i	1.74 ± 0.66b	0.99 ± 0.38d–f	0.75 ± 0.30b–d
	‘Redin’	3.08 ± 1.16d–g	2.84 ± 1.07c–e	0.66 ± 0.26a–d	0.81 ± 0.31c–e
I+Se+SA 10.0	‘Cud Voorburgu’	4.14 ± 0.71g–m	3.54 ± 0.15fg	1.51 ± 0.27gh	1.90 ± 0.14k–m
	‘Zimująca’	5.26 ± 0.76mn	3.62 ± 0.31f–h	1.67 ± 0.12g–k	1.52 ± 0.14ij
	‘Maugli’	5.51 ± 1.09n	4.46 ± 0.61k	1.65 ± 0.20g–j	1.81 ± 0.14jl
	‘Królowa lata’	4.66 ± 1.08i–n	4.26 ± 0.60i–k	1.46 ± 0.19g	2.11 ± 0.12lm
	‘Lollo rossa’	2.93 ± 1.05c–g	2.39 ± 0.91c	1.09 ± 0.44f	0.79 ± 0.31c–e
	‘Redin’	3.08 ± 1.16d–g	3.02 ± 1.14de	1.08 ± 0.42ef	0.87 ± 0.33c–f

*Means in the column followed by different letters for “Treatments × Variety” differ significantly at P < 0.05 (n = 8). d.w., dry weight.*

Adding SA to the media (compared to the cultivation of plants on the nutrient solution without SA) had a different effect on the level of the accumulation of I, Se, SeMet, and SeCys in the leaves and roots of particular lettuce varieties (Tables 1, 2).

#### Effect of SA Upon the I Uptake

Applying SA to media (in a dose of 0.1 mg ⋅ dm^−3^) resulted in significant increases in the accumulation of I in the leaves and roots of plants of the ICEL ‘Królowa lata’ variety (Table 1). In BUTL ‘Zimująca’ and ICEL ‘Maugli’ varieties, only SA applied at the dose of 0.1 mg ⋅ dm^−3^ caused the significant increase in the degree of I accumulation in leaves. In the leaves of the REDL ‘Lollo rossa’ variety, this reaction was found after the application of 10 mg SA ⋅ dm^−3^. Next, in the BUTL ‘Cud Voorburgu’ variety, applying SA in each concentration (0.1, 1.0, and 10 mg SA ⋅ dm^−3^) resulted in a significant decrease in I content in leaves, and a similar reaction was found in the leaves of the ICEL ‘Maugli’ and REDL ‘Redin’ plants after applying SA in a doses of 1.0 and 10 mg SA ⋅ dm^−3^.

In the BUTL ‘Zimująca’ variety, simultaneous significant increases in the degree of I accumulation were observed both in leaves and in roots, after adding to the nutrient solution SA in 0.1 mg ⋅ dm^−3^ dose, when compared with the object with the application of only I+Se. In the remaining tested varieties, the I content in roots did not change significantly as a result of SA application to the media.

#### Effect of SA on the Intake of Se, and the Accumulation of Se-Amino Acid

In all studied varieties if lettuce, adding SA to the nutrient solution, at the dose of 0.1 mg ⋅ dm^−3^ (compared with the application of I+Se alone) resulted in a significant increase of Se in leaves and roots (Table 1). However, after applying SA in two higher doses (1.0 and 10 mg SA ⋅ dm^−3^), the content of Se in the roots and leaves of all varieties remained at the same level or lower as that after applying I+Se without SA. The only exceptions were the increases in Se content in the leaves of the REDL ‘Redin’ variety (after applying 10 mg SA ⋅ dm^−3^) and in the roots of the plants of the variety ICEL ‘Królowa lata’ cultivated on the nutrient solution with the addition of 1.0 mg SA ⋅ dm^−3^.

The application of SA to the nutrient solution in 1 and 10 mg dm^−3^ doses resulted in significant increases of the content of: (1) SeMet in the leaves of varieties BUTL ‘Zimująca,’ ICEL ‘Maugli’ and REDL ‘Lollo rossa’ varieties, while the application of the dose of 10 mg SA ⋅ dm^−3^, in the increases in the content of (2) SeCys in the leaves of both varieties of REDL ‘Redin’ and ‘Lollo rossa’ (Table 2)-compared with the cultivation of the plants on the nutrient solution with I+Se (without SA).

### Content of SA in Lettuce Plants

The content of SA in the leaves and roots of particular lettuce varieties was a relatively variable feature (Table 3). In the case of roots, it was only in the REDL ‘Lollo rossa’ variety where significant (compared with the control and exclusive application of I+Se) increase in SA content in roots resulting from the application of SA to the nutrient solution occurred, which was to the same degree in all three SA dosages used. Regarding the leaves, it was only in the ICEL ‘Maugli’ variety where there was more than a tenfold increase in the content of SA in the leaves of plants growing on the media with 1.0 and 10 mg SA ⋅ dm^−3^ dosages. It was a significant increase of SA content compared with the control, and the treatments with I+Se and I+Se+SA 0.1 mg SA ⋅ dm^−3^.

**Table 3:** Effects of I, Se, and salicylic acid (SA) application on the concentrations of SA and proline in leaves and roots of lettuce.

Treatments	Variety	mg SA ⋅ kg^−1^ d.w.		mg proline ⋅ kg^−1^ d.w.	
		Leaves	Roots	Leaves	Roots
Control	‘Cud Voorburgu’	3.91 ± 0.13i	4.79 ± 0.06ef	162.5 ± 37.9cd	238.1 ± 66.2e–g
	‘Zimująca’	2.63 ± 0.07gh	5.43 ± 0.03gh	294.5 ± 77.9k	225.7 ± 28.7de
	‘Maugli’	6.65 ± 0.44l	2.07 ± 0.17b	287.1 ± 84.8k	241.9 ± 66.4e–g
	‘Królowa lata’	2.81 ± 0.34h	5.83 ± 0.26gh	184.3 ± 56.8ef	171.9 ± 43.2b
	‘Lollo rossa’	0.41 ± 0.07a–c	1.29 ± 0.08a	11.2 ± 4.2a	30.8 ± 11.7a
	‘Redin’	0.17 ± 0.04a	5.43 ± 0.04h	22.8 ± 8.6a	41.6 ± 15.7a
I+Se	‘Cud Voorburgu’	1.13 ± 0.12ef	9.51 ± 0.09L	220.0 ± 57.9gh	270.3 ± 82.2hi
	‘Zimująca’	8.44 ± 0.48m	8.26 ± 0.09k	246.5 ± 64.2ij	261.0 ± 63.9gh
	‘Maugli’	2.96 ± 0.07h	2.33 ± 0.14bc	228.1 ± 70.7hi	222.1 ± 56.6c–e
	‘Królowa lata’	2.02 ± 0.27g	5.88 ± 0.16h	189.2 ± 57.0ef	225.1 ± 53.4de
	‘Lollo rossa’	0.36 ± 0.04ab	2.36 ± 0.14bc	12.8 ± 4.9a	46.1 ± 17.5a
	‘Redin’	1.06 ± 0.18d–e	7.61 ± 0.12j	22.8 ± 8.6a	39.8 ± 15.1a
I+Se+SA 0.1	‘Cud Voorburgu’	1.25 ± 0.13f	7.86 ± 0.46jk	247.0 ± 60.3ij	241.5 ± 63.0e–g
	‘Zimująca’	5.93 ± 0.04k	5.03 ± 0.01fg	326.3 ± 94.7l	332.5 ± 36.6j
	‘Maugli’	2.01 ± 0.58g	4.14 ± 0.41e	214.8 ± 63.0gh	289.1 ± 78.0i
	‘Królowa lata’	0.55 ± 0.04a–e	3.43 ± 0.16d	170.6 ± 48.6de	233.2 ± 45.2ef
	‘Lollo rossa’	0.32 ± 0.03ab	4.73 ± 0.38ef	10.8 ± 4.1a	51.7 ± 19.6a
	‘Redin’	0.87 ± 0.07b–f	7.49 ± 0.17j	26.8 ± 10.1a	39.3 ± 14.9a
I+Se+SA 1.0	‘Cud Voorburgu’	2.73 ± 0.11h	8.28 ± 0.05k	233.7 ± 60.4h–j	236.1 ± 71.9ef
	‘Zimująca’	5.02 ± 0.22j	6.77 ± 0.21i	249.6 ± 70.8ij	250.7 ± 57.7f–h
	‘Maugli’	17.53 ± 0.50no	3.83 ± 0.12de	252.2 ± 77.0j	227.8 ± 55.7d–f
	‘Królowa lata’	0.46 ± 0.10a–d	4.08 ± 0.10e	148.0 ± 42.1bc	159.4 ± 34.9b
	‘Lollo rossa’	0.70 ± 0.03a–f	3.84 ± 0.10de	10.1 ± 3.8a	36.4 ± 13.8a
	‘Redin’	0.69 ± 0.16a–f	8.06 ± 0.37jk	30.2 ± 11.4a	35.4 ± 13.4a
I+Se+SA 10.0	‘Cud Voorburgu’	2.44 ± 0.10gh	7.62 ± 0.17j	191.7 ± 40.2ef	207.1 ± 60.7cd
	‘Zimująca’	5.23 ± 0.31j	5.78 ± 0.11h	201.0 ± 45.1fg	223.8 ± 33.7c–e
	‘Maugli’	12.45 ± 0.16n	2.74 ± 0.12c	255.2 ± 77.5j	201.2 ± 51.1c
	‘Królowa lata’	0.87 ± 0.09b–f	3.95 ± 0.37de	131.5 ± 34.5b	171.2 ± 41.3b
	‘Lollo rossa’	1.02 ± 0.04c–e	4.78 ± 0.12ef	15.3 ± 5.8a	32.6 ± 12.4a
	‘Redin’	1.16 ± 0.01ef	5.92 ± 0.23h	28.7 ± 10.9a	32.2 ± 12.2a

*Means in the column followed by different letters for “Treatments × Variety” differ significantly at P < 0.05 (n = 8). d.w., dry weight.*

Only the leaves of the control plants of BUTL ‘Cud Voorburgu’ and ICEL ‘Królowa lata’ varieties showed significantly higher SA content than that found on the media containing I+Se and an addition of SA in three different doses.

The leaves and roots of BUTL ‘Zimująca’ variety treated only with I+Se contained the greatest amounts of SA, and the content of the acid in the leaves of this variety decreased after the application of SA to the nutrient solution. A similar effect, but only in roots, was found with the fertilization with I+Se, in the form of the greatest accumulation of SA, while adding this compound to the nutrient solution resulted in the decrease of the content of SA in the roots of BUTL ‘Cud Voorburgu’ and ICEL ‘Królowa lata’ varieties.

### Content of Proline in Lettuce Plants

The leaves and roots of REDL ‘Lollo rossa’ and ‘Redin’ varieties showed a few dozen times lower proline content than the remaining varieties of lettuce (Table 3). Furthermore, in these two varieties, the proline content in leaves and roots did not differ at all after adding I, Se, and SA to the media, compared with the control plants.

In the leaves of BUTL ‘Zimująca’ and ICEL ‘Królowa lata’ varieties, adding SA in the two highest doses (1.0 and 10 mg SA ⋅ dm^−3^) resulted in a significant decrease of proline content compared with the application 0.1 mg SA ⋅ dm^−3^.

Regarding the roots, the significant increases in proline content (compared with the control plants, and other tested treatments) were noted only in the roots of plants of BUTL ‘Zimująca,’ ICEL ‘Maugli,’ and ‘Królowa lata’ varieties cultivated on the nutrient solution with the addition of I+Se+SA 0.1 mg ⋅ dm^−3^.

## Discussion

### Plant Biomass and Chemical Composition of Lettuce Plants

The obtained results indicate that four out of six varieties failed to show the negative reaction, in terms of the amount of biomass, to the enriching in I and Se, and to the additional application of SA to the nutrient solution. It confirms that the used doses of I, Se, and SA were safe for the following varieties: BUTL ‘Cud Voorburgu’ and ‘Zimująca,’ ICEL ‘Królowa lata,’ and REDL ‘Lollo rossa.’ It was only in the REDL ‘Redin’ variety where a negative reaction of plants via decreasing their biomass was noted to the application of I+Se and I+Se+SA to the nutrient solution. It is the reason that a remarkable portion of this discussion was devoted to this variety of lettuce, and in the final part of this chapter, the decrease in the biomass of the ICEL ‘Maugli’ variety in response to I+Se+SA 10 mg ⋅ dm^−3^ treatments was commented.

The decrease in the biomass (leaves and whole plants (roots + leaves/heads/) of REDL ‘Redin’ variety was observed in each of four treatments with the cultivation on the nutrient solution enriched in I+Se and I+Se+SA. Therefore, it is likely that the total content of I and Se in the leaves and roots exceeded the threshold of harmfulness for this variety. In our opinion, one can refer only to exceeding the threshold of harmfulness but not to the threshold of toxic content of both elements for the plants of the REDL ‘Redin’ variety. In the plants of this variety, only the decrease in biomass was observed, but none of the symptoms of the toxic effect which are characteristic of excessive I accumulation were observed (Blasco et al., 2008, 2010a,b) and Se (Ríos et al., 2008, 2010; Hawrylak-Nowak, 2013; Esringu et al., 2015). It is interesting to note that, in the second variety of the REDL group, i.e., ‘Lollo rossa,’ the application of I+Se and I+Se+SA (in three different doses) did not result in the decreased the biomass of plants. It can be supposed that the reason behind the different reactions of these two varieties could be the higher Se accumulation in the ‘Redin’ variety than that in the ‘Lollo Rossa’ variety and analogously lower I accumulation. Thus, we conclude that the total level of the I+Se accumulation in REDL ‘Lollo rossa’ plants did not exceed the harmful level, as it was in the case in REDL ‘Redin’ plants.

In roots, immediately after the uptake of SeO_3_^2−^ ion by the plants, occurs conversion of that ion to organo-Se compounds, and principally in this form selenium is transported to leaves where it undergoes further transformations to other organic compounds (Winkel et al., 2015). The intensified synthesis/accumulation of proline (Hayat et al., 2012) and SA (Hayat et al., 2010) in plants is their biochemical response to the occurrence abiotic or biotic stress factors. In plants, the signal molecule associated with stress factors can be SA itself or a volatile ester of this acid, i.e., methyl salicylate (MeSA). MeSA is synthesized by salicylic acid carboxyl methyltransferase /SAMT/ (Tieman et al., 2010). The volatile MeSA, floating in air serves to transfer the biochemical signal between plants, or even between different leaves of the same plant (Taiz and Zeiger, 2010). Apart from the methylation process, SA in plants can undergo many other transformations, e.g., the process of glycosylation, conjugation with amino acids (Dempsey et al., 2011) or be used to the formation of sugar conjugates with the participation of salicylic acid 3-hydroxylase enzyme (Zhang et al., 2013). This enzyme leads, e.g., to the transformation of SA into 2,3-dihydroxybenzoic acid, i.e., into the deactivated form of SA (Hennig et al., 1993; Bartsch et al., 2010).

In the context of the information mentioned above, it is interesting to note that, in the plants of the REDL ‘Redin’ variety, the decrease in the biomass productivity (after the application of I, Se, and SA) was not accompanied by the change in the proline content in leaves and roots. A simultaneous significant increase, compared with the control, in the SA content in leaves and roots of this variety was, however, observed after the application of I+Se. Furthermore, in REDL ‘Redin’ plants cultivated on the nutrient solution with the highest dose of exogenous SA (10 mg SA ⋅ dm^−3^), the highest SA content in leaves was found within that variety, while the SA content in the roots was the same as in the control plants.

The selenocysteine methyltransferase (SMT) enzyme participates in the transformation of SeCys to methyl-SeCys. As a result, the potentially toxic Se-amino acid (SeCys) is transformed into seleno-metabolites, which are less harmful to plants (Ellis et al., 2004). The data from scientific publications indicates that, in the lettuce plants of the BUTL type, the exogenous SA causes the increase in the activity of the gene coding SMT enzyme (Smoleń et al., 2016). As the consequence of the increased SMT activity, the increased intensity of the methylation process of Se can be seen via the synthesis of volatile dimethyl diselenide (DMDSe) and dimethyl selenide (DMSe) (Winkel et al., 2015). For this reason, exogenous SA can cause the decrease in the Se accumulation in the leaves of BUTL-type lettuce plants (Smoleń et al., 2016). In our study, the interaction between SA application (doses 1.0 and 10 versus 0.1 mg SA ⋅ dm^−3^) and the decreased Se accumulation in the leaves of the two lettuce varieties of the BUTL type was identical to the results of the study by Smoleń et al. (2016). However, in the case of the REDL ‘Redin’ variety, the exogenous SA caused the intensified accumulation of Se (but not I) in leaves. In that variety, the exogenous SA probably did not lead to the increase in the degree of the methylation of Se compounds (a mechanism which could lower the Se content in leaves). As a matter of fact, we have not performed the analyses of MeSA gene activity, nor of the levels of DMDSe and DMSe syntheses. However, the results of Se-amino acid determinations (low content of SeMet and SeCys) indicate that, after applying exogenous SA, in both varieties of the REDL type, the majority of the pool of SeO_3_^2−^ ions having been taken up by plants had to stay there in this form or be converted to the organic Se forms other than SeMet and SeCys. Therefore, it can be assumed with the great probability that exogenous SA could slow down the process of the methylation of DMDSe and DMSe in REDL ‘Redin’ plants. We so conclude this because SeMet adn SeCys amino acids are indispensable precursor to the synthesis of these two volatile Se compounds (Winkel et al., 2015). Because of the above fact, after the application of SA, the decrease in the quantity of Se escaping to the atmosphere from the leaves of REDL ‘Redin’ must have occurred. In the light of the above, the REDL ‘Redin’ plants must have experienced a specific drop in the biomass productivity. It probably resulted from the low capacity of this variety to synthesize SeMet and SeCys (and, in consequence, to the lower levels of DMDSe and DMSe syntheses). It resulted in into an increased level of Se accumulation after the application of exogenously higher SA-to reach the level exceeding the harmfulness threshold of Se (more Se and I) for the plants of the REDL ‘Redin’ variety.

As it was already mentioned, SA is a compound responsible for the plant reaction to abiotic and biotic stress factors (Hayat et al., 2010). The results of our study indirectly indicate that in none of the six varieties of lettuce did the exogenously provided SA take part in the protective mechanisms in plants which could be directed at lowering the degree of I and Se accumulation. Quite the opposite, in the studied varieties, SA (reaction specific for the dose of SA) resulted in the increased degree of I and/or Se accumulation in the leaves of lettuce. A specific effect in all varieties was the stimulating effect of the lowest concentration of 0.1 mg SA ⋅ dm^−3^ upon the increase in the Se accumulation in leaves and roots. It could stem from the activation of processes responsible for methylation or the hydrolysis of SA in plants (Hennig et al., 1993; Bartsch et al., 2010; Dempsey et al., 2011; Zhang et al., 2013), at the expense of weakening the processes associated with the syntheses of DMDSe and DMSe. Increasing the efficiency or initiating the above mentioned processes associated with SA metabolism are high energy-consuming. Perhaps, for the lettuce plants, the process of methylation of SA to MeSA is prioritized higher than the process of Se methylation. On the basis of the increased degree of Se accumulation (as an effect of applying SA), we conclude that the lettuce plants of the REDL ‘Redin’ variety can probably commit more energy into the methylation of MeSA than in the process of synthesizing the volatile DMDSe and DMSe compounds.

In the ICEL ‘Maugli’ variety, the relationships between the uptake and metabolic pathways of I, Se, and SA look different than these in the REDL ‘Redin’ variety. The significant decrease in the size of root and head biomasses found in the ICEL ‘Maugli’ variety (only after application of I+Se+SA 10 mg ⋅ dm^−3^) had to be an effect of joint actions of exogenous SA on roots and a simultaneous accumulation of major quantities of SA, I, and Se in leaves. The highest content of SA determined in the leaves of the ICEL ‘Maugli’ variety (in I+Se+SA 1.0 and I+Se+SA 10 treatments) could have been a specific response of this variety to exogenous SA. This reaction could involve the intensified synthesis of this compound in leaves and/or effective transportation of exogenous SA from roots to leaves.

The effect of selenium fertilization can include the increase in the content of soluble sugars in leaves which have been, for example, found in alfalfa /*Medicago sativa* L./ plants (Owusu-Sekyere et al., 2013). Following the application of an I+Se+SA in 10 mg ⋅ dm^−3^ dose, the highest content of SeMet was found in the leaves of the ICEL ‘Maugli’ variety. Within that treatments, also the highest sugar content [the sum of sugars /glucose + fructose + saccharose/ applied as 1000 mg sugars ⋅ 100 g^−1^ f.w. of leaves] as found on average in the leaves, where app. 500–600 mg sugars ⋅ 100 g^−1^ f.w. of leaves compared with the five remaining varieties and tested media (data not presented). In our assessment, the great accumulation of SeMet in the leaves of the ICEL ‘Maugli’ variety (I+Se+SA 10 mg ⋅ dm^−3^) can signify the elevated level of the metabolism of Se-compounds, including the synthesis of volatile DMSe of which SeMet is a precursor. Such interpretation seems to be correct, because the plants needed the energy necessary in the metabolic pathway responsible for the conversion of SeMet to DMSe (Winkel et al., 2015). Again, in the leaves of ICEL ‘Maugli’ lettuce treated with I+Se+SA at doses of 1.0 and 10 mg ⋅ dm^−3^, a remarkable decrease in I content was noted, which probably also was subjected to the energy-consuming processes of volatilisation (methylation) to CH_3_I. The energy needed for the methylation of I and Se compounds is generated in the processes of respiration in cells via oxidizing sugars. The increased consumption of sugars in the methylation processes of I and Se resulted in the unavailability of that energy to the formation of ICEL ‘Maugli’ plant biomass. This way of interpreting results is further enhanced by the fact that, in line with increase in SA concentration in the nutrient solution, a clear drop in Se content (and also, to a certain extent, of SeMet and SeCys content) was found in the roots of ICEL ‘Maugli.’ It indicates that exogenous SA at the dose of 10 mg ⋅ dm^−3^ of the nutrient solution, in ICEL ‘Maugli’ plants had an utmost stimulating effect on the process of the transportation of Se (SeO_3_^−2^ and/or Se-organic compounds) from roots to leaves where it undoubtedly underwent the process of methylation.

The level of proline content in both varieties of in the REDL group was several hundred times lower than in the remaining varieties of BUTL and ICEL. Therefore, we conclude that the cultivation conditions in the greenhouse in autumn/winter seasons were more optimal for both REDL-type varieties than for the other four varieties of lettuce (two types of BUTL and two types of ICEL). The studies of Koudela and Petříková (2008) demonstrated that the yield potential of *Lactuca sativa* L. var. *crispa* L. lettuces (including the varieties ‘Lollo rossa’ and ‘Redin’) depended upon climatic conditions and the timing of cultivation (spring, summer, and autumn). These authors found that the ‘Redin’ variety has a higher yield potential in particular times of cultivation than ‘Lollo rossa.’ Furthermore, the ‘Redin’ was characterized as a variety suitable for autumn outside cultivation. Li et al. (2014) found that *Lactuca sativa* L. var. *crispa* L. is more resistant to abiotic stress factors than other botanical varieties of lettuce.

### Biofortification Target

The percentage of recommended daily allowance for I (RDA-I) and Se (RDA-Se) supplied from one serving of 50 g fresh lettuce leaves was calculated using the results of I and Se content in fresh lettuce leaves as well as the recommended daily intake of these two elements for adults: 150 μg I and 55 μg Se daily (Institute of Medicine, 2000; Andersson et al., 2007).

The effect of the application of I, Se, and SA on the level of I and Se accumulation in leaves (Table 1) had been directly reflected in the calculated level of percentage coverage of the required amounts of RDA-I and RDA-Se for a consumer, resulting from a theoretical consumption of 50 g of fresh leaves of the studied varieties of lettuce (Figure 2A,B). The ratio of the molar content of I:Se in the leaves of particular varieties was a parameter depending on the addition of I, Se, and SA to the nutrient solution (Figure 2C).

The objective of biofortification is to increase the content of mineral components to such quantities as to effectively increase the possibility of covering the feeding allowance of consumers for particular elements in plants (White and Broadley, 2009). The recommended daily allowance (RDA) for I and Se depends on age and sex. For example, in adults, the RDAs for these elements are 150 μg I and 55 μg Se. For pregnant women and breast-feeding mothers, the RDAs are increased and amount to 200–300 μg I and 60–70 μg Se (Institute of Medicine, 2000; Andersson et al., 2007).

In our study, there is a possibility of covering the RDA-A and RDA-Se requirements by such a dose of fresh lettuce leaves. The results of these calculations demonstrated that the process of the biofortification of plants in I and Se effectively increased the possibility of covering the requirements by the RDA-I and RDA-Se lettuces.

The mean percentage value of covering of RDA-I requirement was the lowest in both varieties of ICEL (121.3 and 167.7% for the varieties ‘Maugli’ and ‘Królowa lata,’ respectively), and the highest in the REDL variety ‘Lollo rossa,’ i.e., 348.2% RDA-I. Simultaneously, REDL ‘Lollo rossa’ and BUTL ‘Cud Voorburgu’ showed the highest values of the % RDA-Se index (44.8 and 44.7%, respectively). And both varieties of ICEL were characterized by the lowest values of %RDA-Se, i.e., 23.8% ‘Królowa lata’ and 27.1% ‘Maugli’.

The ratio of I:Se molar content in food is as important as the parameters of RDA-I and RDA-Se. The optimum I:Se molar ratio in the daily food intake for humans, depending on age and sex, calculated on the basis of RDA-I and RDA-Se, stays within 4.4–8.8:1 (Institute of Medicine, 2000; Andersson et al., 2007). The need to maintain an adequate proportion between these elements results, e.g., from the fact that I and Se perform very important roles in the correct function of the thyroid gland. The essence of the relationship of I versus Se in the human (or animal) body consists in the fact that three of iodothyronine deiodinases (D1, D2, and D3) are selenium-dependent enzymes (Bianco and Kim, 2006).

In our study, both ICEL varieties cultivated on the control nutrient solution (non-fortified plants) had the I:Se ratio in leaves, i.e., 10:1 in ‘Królowa lata’ variety and 4.9:1 in ‘Maugli’ variety, that is beneficial to consumers. Nevertheless, the possibilities of covering RDA-I and RDA-Se by both varieties of ICEL derived from the cultivation on the control nutrient solution were insignificant.

The cultivation of plants on the media fortified with I, Se, and SA allowed obtaining the I:Se ration of molar content in leaves at the level approximating that which is optimum for the consumer. So was the case of the ICEL ‘Maugli’ variety, in which the I:Se ratio in leaves was 7.0:1, on average. In the remaining varieties, the calculated I:Se ratio in the leaves of plants fertilized with these elements was slightly higher than the optimum for the consumer. The averages for particular varieties were as follows: 10.9:1 for ICEL ‘Królowa lata,’ 12.1:1 for BUTL ‘Cud Voorburgu,’ 12.2:1 for REDL ‘Redin,’ 14.3:1 for BUTL ‘Zimująca,’ and 18.2:1 for REDL ‘Lollo rossa.’

## Conclusion

Exogenous SA added to the nutrient solution can be considered to be a biostimulating compound improving the efficiency of the uptake of Se by plants (roots and leaves) of six tested varieties of lettuce. This effect was observed in all cultivated varieties after the application combined with the lowest dose of SA, i.e., 0.1 mg dm^−3^, nutrient solution.

Next, the effect of SA on the I accumulation in leaves was a feature specific to the varieties and the dosage of acid used. The positive effects of all dosages of exogenous SA upon the increase in the degree of I accumulation was found in the ICEL ‘Królowa lata’ variety. The lowest dose of 0.1 mg SA ⋅ dm^−3^ nutrient solution increased the effectiveness of the I fortification of leaves in BUTL ‘Zimująca’ and ICEL ‘Maugli’ varieties. In the case of the REDL ‘Lollo rossa’ variety, this effect was obtained with applying SA at the dose of 10 mg dm^−3^. Next, in three varieties BUTL ‘Cud Voorburgu,’ ICEL ‘Maugli,’ and REDL ‘Redin,’ the exogenous SA contributed to the decrease in the I accumulation in leaves.

We conclude that the processes of uptake, transportation, and metabolism of exogenous SA can affect the process of I and Se in plants; however, the direction and intensity of these processes depend on the features of a given variety of lettuce. With great probability, one can assume that the quantitative changes in the accumulation of I, Se, and SA in plants is dictated by the mutual feedback based on the course of the metabolism of I, Se, and SA specific to each of the tested varieties of lettuce.

In our study, the SA and proline amino acid were not specific biochemical markers that could confirm the occurrence of stress conditions caused by the increased degree of I and Se accumulation in lettuce plants.

The REDL ‘Redin’ variety turned out to produce the best yield (forming the largest heads), and it was simultaneously the most susceptible to the decrease in the biomass of plants resulting from the simultaneous addition of I+Se to the nutrient solution, and additionally SA, in each of the applied dosages.

The I:Se ratio of molar content in leaves that is the most optimum for the consumer (7.0:1) was found in the ICEL ‘Maugli’ variety. In the remaining varieties, the I:Se ratio in the leaves of plants fertilized with these elements was slightly higher than is considered to be optimum for the consumer.

## Author Contributions

SS was the leader of the project, co-author of the method of lettuce biofortification with iodine and selenium with additional application of salicylic acid, coordinator of experiment and laboratory analyses, author of the modification method of the determination of iodine (by ICP-OES) as well as SeMet, SeCys and proline by capillary electrophoresis, conducted the analysis of results, and prepared the manuscript. IK was the co-author of the method of lettuce biofortification with iodine and selenium with additional application of salicylic acid, conducted the experiment with the lettuce cultivation, performed the chemical analysis, and helped with preparing the manuscript. PK provided scientific consultations, assistance in developing a research concept, and helped in preparing the manuscript. MH helped in performing the chemical analysis of plant samples and helped with preparing the manuscript. WS supervised the research and helped in preparing the manuscript.

## Conflict of Interest Statement

The authors declare that the research was conducted in the absence of any commercial or financial relationships that could be construed as a potential conflict of interest.

## References

[B1] Andersson M. de Benoist B. Darnton-Hill I. Delange F. (2007). *Iodine Deficiency in Europe: A Continuing Public Health Problem.* Geneva: World Health Organization.

[B2] Bartsch M. Bednarek P. Vivancos P. D. Schneider B. von Roepenack-Lahaye E. Foyer C. H. (2010). Accumulation of isochorismate-derived 2, 3-dihydroxybenzoic 3-O-β-D-xyloside in *Arabidopsis* resistance to pathogens and ageing of leaves. *J. Biol. Chem.* 285 25654–25665. 10.1074/jbc.M109.092569 20538606 PMC2919129

[B3] Bianco A. C. Kim B. W. (2006). Deiodinases: implications of the local control of thyroid hormone action. *J. Clin. Invest.* 116 2571–2579. 10.1172/JCI29812 17016550 PMC1578599

[B4] Blasco B. Rios J. J. Cervilla L. M. Sanchez-Rodrigez E. Ruiz J. M. Romero L. (2008). Iodine biofortification and antioxidant capacity of lettuce: potential benefits for cultivation and human health. *Ann. Appl. Biol.* 152 289–299. 10.1111/j.1744-7348.2008.00217.x

[B5] Blasco B. Rios J. J. Cervilla L. M. Sanchez-Rodriguez E. Rubio-Wilhelmi M. M. Rosales M. A. (2010a). Photorespiration process and nitrogen metabolism in lettuce plants (*Lactuca sativa* L.)*:* induced changes in response to iodine biofortification. *J. Plant Growth Regul.* 29 477–486. 10.1007/s00344-010-9159-7

[B6] Blasco B. Rios J. J. Leyva R. Cervilla L. M. Sanchez-Rodriguez E. Rubio-Wilhelmi M. (2010b). Does iodine biofortification affect oxidative metabolism in lettuce plants? *Biol. Trace Elem. Res.* 142 831–842. 10.1007/s12011-010-8816-9 20838926

[B7] Cakmak I. (2008). Enrichment of cereal grains with zinc: agronomic or genetic biofortification? *Plant Soil* 302 1–17. 10.1007/s11104-007-9466-3

[B8] Dai J. L. Zhu Y. G. Huang Y. Z. Zhang M. Song J. L. (2006). Availability of iodide and iodate to spinach (*Spinacia oleracea* L.) in relation to total iodine in soil solution. *Plant Soil* 286 301–308. 10.1007/s11104-006-9139-7

[B9] Dayod M. Tyerman S. D. Leigh R. A. Gilliham M. (2010). Calcium storage in plants and the implications for calcium biofortification. *Protoplasma* 247 215–231. 10.1007/s00709-010-0182-0 20658253

[B10] Dempsey D. A. Vlot A. C. Wildermuth M. C. Klessing D. F. (2011). Salicylic acid biosynthesis and methabolism. *Arabidopsis Book* 9:e0156. 10.1199/tab.0156 22303280 PMC3268552

[B11] Ellis D. R. Sors T. G. Brunk D. G. Albrecht C. Orser C. Lahner B. (2004). Production of Se-methylselenocysteine in transgenic plants expressing selenocysteine methyltransferase. *BMC Plant Biol.* 4:1. 10.1186/1471-2229-4-1 15005814 PMC343276

[B12] Esringu A. Ekinci M. Usta S. Turan M. Dursun A. Ercisli S. (2015). Selenium supplementation affects the growth, yield and selenium accumulation in lettuce (*Lactuca sativa* L.). *C. R. Acad. Bulg. Sci.* 68 801–810.

[B13] García-Bañuelos M. L. Sida-Arreola J. P. Sánchez E. (2014). Biofortification -promising approach to increasing the content of iron and zinc in staple food crops. *J. Elem.* 19 865–888. 10.5601/jelem.2014.19.3.708

[B14] Golubkina N. A. Kosheleva O. V. Krivenkov L. V. Dobrutskaya H. G. Nadezhkin S. Caruso G. (2017). Intersexual differences in plant growth, yield, mineral composition and antioxidants of spinach (*Spinacia oleracea* L.) as affected by selenium form. . *Sci. Hort.* 225 350–358. 10.1016/j.scienta.2017.07.001

[B15] Gonzali S. Kiferle C. Perata P. (2017). Iodine biofortification of crops: agronomic biofortification, metabolic engineering and iodine bioavailability. *Curr. Opin. Biotech.* 44 16–26. 10.1016/j.copbio.2016.10.004 27835794

[B16] Hawrylak-Nowak B. (2013). Comparative effects of selenite and selenate on growth and selenium accumulation in lettuce plants under hydroponic conditions. *Plant Growth Regul* 70 149–157. 10.1007/s10725-013-9788-5

[B17] Hawrylak-Nowak B. Matraszek R. Pogorzelec M. (2015). The dual effects of two inorganic selenium forms on the growth, selected physiological parameters and macronutrients accumulation in cucumber plants. *Acta Physiol. Plant.* 37:41 10.1007/s11738-015-1788-9

[B18] Hayat Q. Hakat S. Irfan M. Ahmad A. (2010). Effect of exogenous salicylic acid under changing environment: a review. *Environ. Exp. Bot.* 68 14–25. 10.1016/j.envexpbot.2009.08.005

[B19] Hayat S. Hayat Q. Alyemeni M. N. Wani A. S. Pichtel J. Ahmad A. (2012). Role of proline under changing environments A review. *Plant Signal. Behav.* 7 1456–1466. 10.4161/psb.21949 22951402 PMC3548871

[B20] Hegedüsová A. Mezeyová I. Hegedüs O. Andrejiová A. Juríková T. Golian M. (2017). Increasing of selenium content and qualitative parameters in garden pea (*Pisum sativum* L.) after its foliar application. *Acta Sci. Pol. Hort. Cultus.* 16 3–17. 10.24326/asphc.2017.6.1

[B21] Hennig J. Malamy J. Grynkiewicz G. Indulski J. Klessig D. F. (1993). Interconversion of the salicylic acid signal and its glucoside in tobacco. *Plant J.* 4 593–600. 10.1046/j.1365-313X.1993.04040593.x 8252063

[B22] Institute of Medicine (2000). *Dietary Reference Intakes for Vitamin C, Vitamin E, Selenium, and Carotenoids.* Washington, DC: National Academies Press. 25077263

[B23] Jerše A. Maršić N. K. Kroflič A. Germ M. Šircelj H. Stibilj V. (2018). Is foliar enrichment of pea plants with iodine and selenium appropriate for production of functional food? *Food Chem.* 267 368–375. 10.1016/j.foodchem.2018.02.112 29934180

[B24] Kopsell D. A. Kopsell D. E. (2007). “Selenium,” in *Handbook of Plant Nutrition*, eds Barker A. V. Pilbeam D. J. (Boca Raton, FL: CRC Press), 515–549.

[B25] Korobova E. (2010). Soil and landscape geochemical factors which contribute to iodine spatial distribution in the main environmental components and food chain in the central Russian plan. *J. Geochem. Explor.* 107 180–192. 10.1016/j.gexplo.2010.03.003

[B26] Koudela M. Petříková K. (2008). Nutrients content and yield in selected cultivars of leaf lettuce (*Lactuca sativa* L. var. crispa). *Hort. Sci.* 35 99–106. 10.17221/3/2008-HORTSCI

[B27] Krzepiłko A. Zych-Wȩżyk I. Molas J. Skwaryło-Bednarz B. Świȩciło A. Skowrońska M. (2016). The effect of iodine biofortification on selected biological quality parameters of lettuce and radish seedlings. *Acta Sci. Pol. Hortorum Cultus* 15 3–16.

[B28] Lawson P. G. Daum D. Czauderna R. Meuser H. Härtling J. W. (2015). Soil versus foliar iodine fertilization as a biofortification strategy for field-grown vegetables. *Front. Plant Sci.* 6:450. 10.3389/fpls.2015.00450 26157445 PMC4477264

[B29] Li C. C. Dang F. Cang L. Zhou C. F. Zhou D. M. (2014). Integration of metal chemical forms and subcellular partitioning to understand metal toxicity in two lettuce (*Lactuca sativa* L.) cultivars. *Plant Soil* 384 201–212. 10.1007/s11104-014-2194-6

[B30] Li R. Li D. W. Liu H. P. Hong C. L. Song M. Y. Dai Z. X. (2017). Enhancing iodine content and fruit quality of pepper (*Capsicum annuum* L.) through biofortification. *Sci. Hortic.* 214 165–173. 10.1016/j.scienta.2016.11.030

[B31] Mandal S. Mallick N. Mitra A. (2009). Salicylic acid-induced resistance to *Fusarium oxysporum* f. sp. lycopersici in tomato. *Plant Physiol. Biochem.* 47 642–649. 10.1016/j.plaphy.2009.03.001 19329332

[B32] Medrano-Macías J. Leija-Martínez P. González-Morales S. Juárez-Maldonado A. Benavides-Mendoza A. (2016). Use of iodine to biofortify and promote growth and stress tolerance in crops. *Front. Plant Sci.* 7:1146. 10.3389/fpls.2016.01146 27602033 PMC4993787

[B33] Osmić A. Golob A. Germ M. (2017). The effect of selenium and iodine on selected biochemical and morphological characteristics in kohlrabi sprouts (*Brassica oleracea* L. var. *gongylodes L.). Acta Biol*. *Sloven. Ljub.* 60 41–51.

[B34] Owusu-Sekyere A. Kontturi J. Hajiboland R. Rahmat S. Aliasgharzad N. Hartikainen H. (2013). Influence of selenium (Se) on carbohydrate metabolism, nodulation and growth in alfalfa (*Medicago sativa* L.). *Plant Soil* 373 541–552. 10.1007/s11104-013-1815-9

[B35] PN-EN 15111:2008 (2008). *Foodstuffs – Determination of Trace Elements – Determination of Iodine by ICP-MS (Inductively Coupled Plasma Mass Spectrometry).* Warsaw: Polish Committee of Standardization.

[B36] Przybysz A. Wrochna M. Małecka-Przybysz M. Gawrońska H. Gawroński S. W. (2016a). Enrichment of some leafy vegetables with magnesium. *J. Elem.* 21 797–809. 10.5601/jelem.2015.20.4.999

[B37] Przybysz A. Wrochna M. Małecka-Przybysz M. Gawrońska H. Gawroński S. W. (2016b). The effects of Mg enrichment of vegetable sprouts on Mg concentration, yield and ROS generation. *J. Sci. Food Agric.* 96 3469–3476. 10.1002/jsfa.7530 26564475

[B38] Ríos J. J. Blasco B. Cervilla L. M. Rubio-Wilhelmi M. M. Rosales M. A. Sánchez-Rodríguez E. (2010). Nitrogen-use efficiency in relation to different forms and application rates of Se in lettuce plants. *J. Plant Growth Reg.* 29 164–170. 10.1007/s00344-009-9130-7

[B39] Ríos J. J. Rosales M. A. Blasco B. Cervilla L. M. Romero L. Ruiz J. M. (2008). Biofortification of Se and induction of the antioxidant capacity in lettuce plants. *Sci. Hortic.* 116 248–255. 10.1016/j.scienta.2008.01.008

[B40] Rożek S. Myczkowski J. Sady W. Wojtaszek T. (1989). The effect of some factors on the content of nitrate and nitrite in lettuce leaves grown with the nutrient film technique. *I*. The effect of light and growth regulators. *Folia Hort.* 1 31–43.

[B41] Sady W. Rożek S. Gregorczyk J. (1990). The effect of fertilization with different forms of nitrogen on yield and nitrate metabolism in leaves of greenhouse lettuce. I. Yield and content of selected components in lettuce leaves. *Folia Hort.* 2 65–76.

[B42] Smoleń S. Kowalska I. Czernicka M. Halka M. Kȩska K. Sady W. (2016). Iodine and selenium biofortification with additional application of salicylic acid affects yield, selected molecular parameters and chemical composition of lettuce plants (*Lactuca sativa* L. var. *capitata)*. *Front. Plant Sci.* 7:1553. 10.3389/fpls.2016.01553 27803709 PMC5067578

[B43] Smoleń S. Wierzbińska J. Sady W. Kołton A. Wiszniewska A. Liszka-Skoczylas M. (2015). Iodine biofortification with additional application of salicylic acidaffects yield and selected parameters of chemical composition oftomato fruits (*Solanum lycopersicum* L.). *Sci. Hortic.* 188 89–96. 10.1016/j.scienta.2015.03.023

[B44] Spletzer M. E. Enyedi A. J. (1999). Salicylic acid induces resistance to *Alternaria solani* in hydroponically grown tomato. *Phytopathology* 89 722–727. 10.1094/PHYTO.1999.89.9.722 18944699

[B45] Taiz L. Zeiger E. (2010). *Plant Physiology.* Redwood City, CA: The Benjamin Cummings Publishing Company.

[B46] Tari I. Csiszár J. Szalai G. Horváth F. Pécsváradi A. Kiss G. (2002). Acclimation of tomato plants to salinity stress after a salicylic acid pre-treatment. *Acta Biol. Szeged.* 46 55–56.

[B47] Tieman D. Zeigler M. Schmelz E. Taylor M. G. Rushing S. Jones J. B. (2010). Functional analysis of a tomato salicylic acid methyl transferase and its role in synthesis of the flavor volatile methyl salicylate. *Plant J.* 62 113–123. 10.1111/j.1365-313X.2010.04128.x 20070566

[B48] Voogt W. Holwerda H. T. Khodabaks R. (2010). Biofortification of lettuce (*Lactuca sativa* L.) with iodine: the effect of iodine form and concentration in the nutrient solution on growth, development and iodine uptake of lettuce grown in water culture. *J. Sci. Food Agric.* 90 906–913. 10.1002/jsfa.3902 20355129

[B49] Voogt W. Jackson W. A. (2010). Perchlorate, nitrate, and iodine uptake and distribution in lettuce (*Lactuca sativa* L.) and potential impact on background levels in humans. *J. Agric. Food Chem.* 58 12192–12198. 10.1021/jf101227d 21047133

[B50] White P. J. Broadley M. R. (2009). Biofortification of crops with seven mineral elements often lacking in human diets – iron, zinc, copper, calcium, magnesium, selenium and iodine. *New Phytol.* 182 49–84. 10.1111/j.1469-8137.2008.02738.x 19192191

[B51] Winkel L. H. Vriens B. Jones G. D. Schneider L. S. Pilon-Smits E. Bañuelos G. S. (2015). Selenium cycling across soil-plant-atmosphere interfaces: a critical review. *Nutrients* 7 4199–4239. 10.3390/nu7064199 26035246 PMC4488781

[B52] Zhang K. Halitschke R. Yin C. Liu C. J. Gan S. S. (2013). Salicylic acid 3-hydroxylase regulates *Arabidopsis* leaf longevity by mediating salicylic acid catabolism. *Proc. Natl. Acad. Sci. U.S.A.* 110 14807–14812. 10.1073/pnas.1302702110 23959884 PMC3767541

[B53] Zhao F. J. McGrath S. P. (2009). Biofortification and phytoremediation. *Curr. Opin. Plant Biol.* 12 373–380. 10.1016/j.pbi.2009.04.005 19473871

[B54] Żuk-Gołaszewska K. Żerańska A. Krukowska A. Bojarczuk J. (2016). Biofortification of the nutritional value of foods from the grain of *Triticum durum* Desf. by an agrotechnical method: a scientific review. *J. Elem.* 21 963–975. 10.5601/jelem.2015.20.4.950

